# Evaluation of *Artemia* *franciscana* Cysts to Improve Diets for Mass Rearing *Stethorus* *gilvifrons*, a Predator of *Tetranychus* *turkestani*

**DOI:** 10.3390/insects12070632

**Published:** 2021-07-13

**Authors:** Jafar Ebrahimifar, Parviz Shishehbor, Arash Rasekh, Seyed Ali Hemmati, Eric W. Riddick

**Affiliations:** 1 Department of Plant Protection, Faculty of Agriculture, Shahid Chamran University of Ahvaz, Ahvaz 61357-43311, Iran; jafar.ebrahimi@alumni.ut.ac.ir (J.E.); pshishehbor@scu.ac.ir (P.S.); a.rasekh@scu.ac.ir (A.R.); sa.hemmati@scu.ac.ir (S.A.H.); 2 National Biological Control Laboratory, Agricultural Research Service, United States Department of Agriculture, Stoneville, MS 38776, USA

**Keywords:** biological control, Coccinellidae, mass rearing, predator, spider mite, *Tetranychidae*

## Abstract

**Simple Summary:**

The ladybird beetle *Stethorus gilvifrons* is a native predator of spider mites in the Mediterranean region that could be mass-reared and released to control spider mite populations on crop plants. The aim of this research was to test the hypothesis that brine shrimp cysts can improve diets for mass rearing of *S*. *gilvifrons* in the absence of prey, i.e., spider mites. The diet treatments included brine shrimp cysts alone (D1), brine shrimp cysts plus a vitamin B complex (D2), brine shrimp cysts plus date palm pollen (D3), or brine shrimp cysts plus date palm pollen and Mediterranean flour moth eggs (D4). The results revealed that only two diets, D3 and D4, supported predator development to the adult stage and reproduction. The predator reproductive rate and life table estimates indicated that D4 was superior. In conclusion, only a mixed diet of brine shrimp cysts, date palm pollen, and flour moth eggs is suitable for mass rearing *S*. *gilvifrons*.

**Abstract:**

*Stethorus* *gilvifrons* is an acarophagous coccinellid distributed in the Mediterranean region and could potentially be mass-reared for the augmentative biological control of *Tetranychus* *turkestani* and related species on crop plants. The hypothesis that brine shrimp *Artemia franciscana* cysts can improve diets for rearing of *S*. *gilvifrons* was tested in laboratory experiments. The diet treatments included *A*. *franciscana* cysts (D1), *A*. *franciscana* cysts plus a vitamin B complex (D2), *A*. *franciscana* cysts plus date palm pollen (D3), and *A*. *franciscana* cysts plus date palm pollen and *Ephestia* *kuehniella* eggs (D4). The results indicated that D1 did not support immature development. D2 supported egg–larval development but not pupal–adult development. Both D3 and D4 supported development to the adult stage and reproduction. However, D4 was the most effective diet, determined by observations of *S*. *gilvifrons* oviposition behavior and fecundity. A life table analysis corroborated these results; an intrinsic rate of increase, net and gross reproductive rates, and mean generation time were best for *S*. *gilvifrons* fed D4 rather than D3. A mixed diet composed of *A*. *franciscana* cysts, date palm pollen, and *E*. *kuehniella* eggs can be used to mass rear *S*. *gilvifrons*.

## 1. Introduction

The spider mite *Tetranychus turkestani* (Ugarov and Nikolskii) (Acari: *Tetranychidae*) is a major pest of numerous crop plants belonging to over 15 families [1,2,3,4]. It is distributed in Africa, Asia, Europe, and North America [5,6,7,8]. In Southwestern Iran, *T. turkestani* often infests plant species of Solanaceae [9], Fabaceae (Leguminosae) [10], and Cucurbitaceae [11]. Its rapid development and high reproductive capacity can cause significant injury to plants, resulting in a decline in crop yield, if control measures are not implemented [9,10,11,12].

Due to the rapid development of resistance of tetranychids to acaricides [13,14], nonchemical alternative methods of controlling *T*. *turkestani* are being pursued. Biological control using specialist predators in the tribe Stethorini (Coleoptera: Coccinellidae) could be a promising alternative if mass rearing and augmentative releases of one or more species could control tetranychids, such as *T*. *turkestani*, under semi-field conditions in greenhouses or high tunnels or in open agricultural fields [15,16,17].

One promising candidate for mass rearing and augmentative releases is *Stethorus gilvifrons* (Mulsant) (Coleoptera: Coccinellidae). Its proclivity for attacking and consuming tetranychids, including *T*. *turkestani* immature and adult stages, has been demonstrated in previous studies [18,19,20,21]. Preliminary research to develop cost-effective methods to mass rear *S*. *gilvifrons* has provided evidence, for the first time, that *S*. *gilvifrons* can complete development and reproduce on a diet devoid of natural prey, i.e., tetranychid mites. Factitious diets containing eggs of the Mediterranean flour moth *Ephestia kuehniella* Zeller (Lepidoptera: Pyralidae) and date palm *Phoenix dactylifera* L. (Arecales: Arecaceae) pollen supported the development and limited reproduction of *S*. *gilvifrons* in lieu of *T*. *turkestani* [22]. In a follow-up study, *S*. *gilvifrons* was successfully reared on an artificial diet based on vertebrate protein, hen’s egg yolk, and *E*. *kuehniella* eggs [23]. The addition of 2,4-dihydroxybenzioc acid, a potential oviposition stimulant, to this diet significantly improved the reproductive output [23].

Since *E*. *kuehniella* eggs are expensive, this study was initiated to determine the suitability of a less expensive factitious food, i.e., decapsulated cysts (eggs) of brine shrimp *Artemia franciscana* Kellogg (Anostraca: Artemiidae) for *S*. *gilvifrons*. *A*. *franciscana* cysts are two-fold less expensive to produce than *E*. *kuehniella* eggs [24]. Several aphidophagous coccinellids can develop and produce some progeny on a diet of *A*. *franciscana* cysts. For example, *Coleomegilla maculata* DeGeer (Coleoptera: Coccinellidae) developed to the adult stage and the females produced some progeny when reared on a diet solely of *A*. *franciscana* decapsulated cysts [25]. Yet, progeny production improved significantly when *C*. *maculata* females were fed a diet containing a fatty acid (palmitic acid) and *A*. *franciscana* eggs, which had been pulverized into a fine powder [26]. Note that diets containing a mixture of plant products, e.g., bee pollen, and *A*. *franciscana* cysts are more nutritious than *A*. *franciscana* cysts alone for rearing another coccinellid, *Adalia bipunctata* (L.) [27].

In this study, the hypothesis that *A*. *franciscana* cysts can improve diets for *S*. *gilvifrons* was tested. The objectives were to test the diet treatments, including *A*. *franciscana* cysts (D1), *A*. *franciscana* cysts plus a vitamin B complex (D2), *A*. *franciscana* cysts plus date palm pollen (D3), and *A*. *franciscana* cysts plus date palm pollen and *E. kuehniella* eggs (D4) on development and reproduction of *S*. *gilvifrons*. A life table analysis was conducted to further define which diet mixture was most nutritious for *S*. *gilvifrons*.

## 2. Materials and Methods

### 2.1. Stethorus gilvifrons Colony

The initial population of *S. gilvifrons* was originally collected from a sugarcane field (Amir-Kabir Agro-Industry Co., Ahvaz, Iran) around Khuzestan Province in Ahvaz (coordinates, 31°19′13″ N, 48°40′09″ E), Iran. Preadults and adults of *S. gilvifrons* were cultured in separate containers at controlled environmental conditions (25 ± 1 °C, 65% ± 5% RH, and 16-h photophase). The *S. gilvifrons* colony was maintained for several consecutive generations on factitious diets, including mixtures of *A. fransciscana* cysts, date palm pollen, and *E. kuehniella* eggs. The identity of *S. gilvifrons* was confirmed by H. Hodek for a previous study [22]. Voucher specimens were deposited in the insect collection of Shahid Chamran University of Ahvaz, Ahvaz, Iran.

### 2.2. Preparation of Factitious Diets

The initial colony of *E. kuehniella* was purchased from the Golestan Mooud Insectary Company, Ahvaz, Iran and reared continuously based on the method described previously [28]. Eggs were collected daily and stored in a refrigerator at 4 °C for less than two weeks before use. Date palm pollen sheaths were purchased from a rural supplier in Ahvaz City at a cost of 3.0–3.5 USD per kg (equivalent to four sheaths). Approximately 6 g of pollen grains were contained in each kg of sheath. Pollen grains were removed from the sheaths and stored in a freezer at −18 °C before use. A vitamin B complex (BECOVIT^®^) was purchased from a drugstore in Ahvaz.

Capsulated *A. franciscana* cysts were originally prepared by the Aquatics Department, Faculty of Veterinary, Shahid Chamran University of Ahvaz. To decapsulate the cysts, 1 g of *A. franciscana* cysts was weighed and added to 1-L distilled water, and then, 10 g of sodium chloride was added to achieve a 1% salinity. Next, 3 mL of 5% sodium hypochlorite was added to solution, and the cysts were ventilated using an oxygen pump. The decapsulated *A*. *franciscana* cysts were leached and screened-out of the solution using a mesh screen (60 µ, 250 mesh), then stored in a hydrated state in a refrigerator until experimentation.

Decapsulated *A*. *franciscana* cysts were removed from the distilled water before the final preparation for experiments. The factitious diets tested in this study were as follows: *A. franciscana* cysts (0.5 g), *A. franciscana* cysts + vitamin B complex (0.5 g + 1 mL), *A. franciscana* cysts + date palm pollen (0.5 g + 0.04 g), and *A. franciscana* cysts + date palm pollen + *E. kuehniella* eggs (0.5 g + 0.04 g + 0.5 g). A ceramic mortar and pestle were used to mix the diet components, except *E*. *kuehniella* eggs, until well-blended. Then, the *E. kuehniella* eggs were gently mixed with the *A*. *franciscana* cysts/date palm pollen blend. Finally, the diet compositions were transferred to microtubes and stored in a freezer at –18 °C until experimentation.

### 2.3. Experimental Setup

The experiment compared the following diet treatments: *A*. *franciscana* cysts (D1), *A*. *franciscana* cysts plus a vitamin B complex (D2), *A*. *franciscana* cysts plus date palm pollen (D3), and *A*. *franciscana* cysts plus date palm pollen and *Ephestia kuehniella* eggs (D4). The developmental time, survival, adult body weight, adult periods (pre- and postoviposition), fecundity, sex ratio, and egg hatch of *S. gilvifrons* fed different diets were assessed. Using a completely randomized design, first instars of *S. gilvifrons* (˂24 h) from the stock colony were transferred to Petri dishes (6-cm diameter, 1.6-cm length) in a cohort of 3 (*n* = 25) and supplied ad libitum with factitious diet at the base of each Petri dish on a metal net wadding on cotton strands. At least 30 mg of each factitious diet was always present in all Petri dish arenas. The diets were refreshed every two days; the old diet and feces were discarded. Newly emerged adults were sexed and weighed using an N-202 precise digital balance. Female and male adults from the same larval diet were paired and provided one of the diets in the individual Petri dishes (*n* = 15 for each treatment). Metal net wadding was utilized as an oviposition substrate in the factitious diet bioassays. Under the metal net wadding, cotton strands were used. Oviposition substrates were checked daily for eggs to determine the preoviposition period. The oviposition rate (daily fecundity) was monitored during the entire lifespan of the females. All eggs were transferred to new Petri dishes until adult emergence, and the number of male and female *S*. *gilvifrons* adults was recorded to determine sex ratio (proportion of females). The longevity of males and females was also determined. All experiments were conducted under controlled conditions (30 ± 1 °C, 65% ± 5% RH, and 16-h photophase).

### 2.4. Statistical Analysis

The datasets were first verified for normal distribution by the Kolmogorov–Smirnov test. Data on the immature survival and developmental time were analyzed using one-way analysis of variance (one-way ANOVA). Data on the adult body weight, preoviposition period, oviposition period, postoviposition period, adult longevity, total fecundity, sex ratio, and egg hatchability of *S. gilvifrons* reared on different diets were analyzed using a Student’s *t*-test. Data on the immature survival rate and egg hatch rate were arcsine-transformed prior to analysis. Means were separated using Tukey’s HSD test after the one-way ANOVA at the *p* ≤ 0.05 significance level [29].

As described in a previous study [23], an age-stage, two-sex life table procedure [30] was used to analyze the data in response to the development rate among the individuals and stages of development in the diet treatments. Population growth parameters such as the net reproductive rate (*R_o_*), gross reproductive rate (*GRR*), intrinsic rate of natural increase (*r*), finite rate of increase (*λ*), mean generation time (*T*), and doubling time (*DT*) were calculated using the TWOSEX-MSChart program [31]. The bootstrap technique was used to determine the standard errors of the population growth parameters and multiple comparisons were made by the paired bootstrap test with 100,000 samples.

## 3. Results

### 3.1. Immature Development and Survival

A diet of purely *A*. *franciscana* cysts (D1) did not support the development of *S*. *gilvifrons*. Thus, this data was not included in the statistical analysis (Table 1). The survival rate of all immature stages combined did not differ significantly amongst the other three diets (F = 1.42, df = 2, 8; *p* = 0.313). The three diets in the analysis were *A*. *franciscana* cysts plus a vitamin B complex (D2), *A*. *franciscana* cysts plus date palm pollen (D3), and *A*. *franciscana* cysts plus date palm pollen and *E*. *kuehniella* eggs (D4). The development time was significantly shorter for males reared on D2 than on D3 or D4 (males: F = 3.98, df = 2, 18; *p* = 0.039) but not for females (F = 1.55, df = 2, 22; *p* = 0.234; Table 1). Note that none of the *S*. *gilvifrons* immatures reared on D2 survived to the pupal stage. The adult body weights of *S*. *gilvifrons* males and females did not differ significantly between the two diets D3 and D4 (male: *t* = 0.159; df = 44, *p* = 0.874; female: *t* = 0.187, df = 44, *p* = 0.853; Table 1).

### 3.2. Adult Longevity and Fecundity

The longevity and fecundity of *S*. *gilvifrons* adults differed significantly between the diet treatments (Table 2). Males and females lived slightly longer when fed D4 compared to D3 (males, *t* = 3.93, df = 26, *p* ˂ 0.05; females, *t* = 3.46, df = 33, *p* ˂ 0.05). The preoviposition time period did not differ significantly between the diet treatments (*t* = 1.64, df = 33, *p* = 0.125). The oviposition time period did, in fact, differ between the diet treatments (*t* = 4.76, df = 33, *p* ˂ 0.001); females deposited more eggs when fed D4 than D3 (Table 2). The postoviposition period was not influenced by diet (*t* = 1.21, df = 33, *p* = 0.235). The total fecundity, i.e., eggs laid per female over the lifespan, was significantly different (*t* = 4.72, df = 33, *p* ˂ 0.001); fecundity was greater when females were fed D4 compared to D3. The egg hatch rate was greater in D4 than D3 (*t* = 3.43, df = 4, *p* = 0.026). The diet treatments did not affect the sex ratio of the progeny (*t* = 2.07, df = 4, *p* = 0.106).

### 3.3. Life Table Assessment

The diets had significant effects on several life table parameters of *S*. *gilvifrons* (Table 3). The intrinsic rate of a natural increase (*r*) was greater with D4 than D3; the finite rate of increase (λ) was not affected significantly. The net reproductive rate (*R_o_*) and gross reproductive rate (*GRR*) were significantly greater with D4 than D3. The mean generation time (*T*) was significantly less with D4 than D3, but the doubling time was unaffected by the diets.

## 4. Discussion

The failure of *S*. *gilvifrons* to complete development and reproduce when fed a diet of *A*. *franciscana* cysts with or without a vitamin B complex suggests that this diet lacked essential nutrients. However, *S*. *gilvifrons* was capable of developing to the adult stage and producing a limited number of progeny when fed a mixture of *A*. *franciscana* cysts and date palm pollen. This observation suggests that essential nutrients lacking in *A*. *franciscana* cysts are found in date palm pollen. In a recent study, *S*. *gilvifrons* completed development and produced more progeny when fed a mixture of *E*. *kuehniella* eggs plus date palm pollen rather than maize pollen or bee pollen [22]. Date palm pollen contains fatty acids, amino acids, flavonoids, saponins, sterols, and other components and has been reputed to improve fertility in humans [32]. Perhaps the content of essential nutrients, e.g., fatty acids, is greater in date palm pollen than in maize or bee pollen. More research is necessary to confirm this supposition.

The observation that a mixed diet of date palm pollen, *A*. *franciscana* cysts, and *E*. *kueniella* eggs was more nutritious than a diet with date palm pollen and *A*. *franciscana* cysts suggests that *E*. *kuehniella* provides essential nutrients not found in the other two components. The soluble protein content [25] and fatty acid content [33] are greater in *E*. *kuehniella* eggs than *A*. *franciscana* cysts, which could partially explain the results herein. Although a treatment composed of *E*. *kueniella* eggs only was not included in this study, a previous work indicated that a diet of *E*. *kuehniella* alone was less nutritious than natural prey, *T*. *turkestani* eggs, for *S*. *gilvifrons* [22].

Comparing the reproductive performance of *S*. *gilvifrons* fed optimal mixed diets in two closely rated studies versus this study suggests that the *A*. *franciscana*, *E*. *kuehniella*, and date palm pollen diet was superior. In other words, the total fecundity, net reproductive rate (*R_o_*), and gross reproductive rate (*GRR*) were 59.72 eggs per female, 29.86, and 46.79, respectively, in this study. Yet, when fed a *E*. *kuehniella*, hen’s egg yolk, and 2,4-DHBA diet, the *S*. *gilvifrons* total fecundity, *R_o_*, and *GRR* were 42.09 eggs per female, 16.30, and 32.38, respectively [23]. When fed a *E*. *kuehniella* and date palm pollen diet, the total fecundity, *R_o_*, and *GRR* were 40.62 eggs per female, 21.48, and 30.08, respectively [22]. A follow-up study comparing these three mixed diets in the same experimental design would confirm the superiority of the *E*. *kuehniella*, *A*. *franciscana*, and date palm pollen diet.

In terms of cost efficiency, eliminating or reducing the quantity of *E*. *kuehniella* eggs in a mixed diet for *S*. *gilvifrons* would represent significant savings. Therefore, more research to identify natural products or other factitious foods of high quality to support the reproduction of *S*. *gilvifrons* in a mass rearing system is encouraged. One possibility is the use of black soldier fly *Hermetia illucens* (L.) (Diptera: Stratiomyidae) larval hemolymph or larval powder as a replacement for *E*. *kuehniella*. *H*. *illucens* has a high protein (amino acid) content. Previous research suggested that larval hemolymph was a good substitute for vertebrate protein (hen’s egg yolk) in an artificial diet for a phytoseiid mite, *Amblyseius swirskii* (Athias–Henriot), an important predator of tetranychid mites [34]. The utilization of *H*. *illucens* larval powder in diets for predatory insects or mites has not been reported in the literature.

In conclusion, this study indicated that *A*. *franciscana* cysts alone are not an adequate diet for *S*. *gilvifrons*, but mixing them with date palm pollen and *E*. *kuehniella* eggs does, in fact, support growth, development, and moderate reproduction. Future research should continue the search for inexpensive natural products or factitious foods that could replace *E*. *kuehniella* eggs in a mixed diet. Rearing over multiple generations without natural prey will be necessary to fully understand the effects of the factitious diet components on the health of *S*. *gilvifrons* and its subsequent capacity to recognize live prey, e.g., *T*. *turkestani*, on crop plants in greenhouses, high tunnels, or open fields upon augmentative release.

## Figures and Tables

**Table 1:** Mean ± SE of the immature survival, developmental time, and adult body weights of *S*. *gilvifrons* fed four different diets containing *A*. *franciscana* cysts.

* Diet	Immature Survival † (%)	Egg–Pupal Development Time (Days) †		Adult Body Weight (mg) ‡	
		Male	Female	Male	Female
D1	-	-	-	-	-
D2	56.91 ± 7.85 ^a^ (*n* = 3)	10.99 ± 1.50 ^b^ (*n* = 5)	11.89 ± 1.50 ^a^ (*n* = 5)	-	-
D3	63.13 ± 3.32 ^a^ (*n* = 3)	13.20 ± 1.40 ^a^ (*n* = 5)	13.83 ± 1.92 ^a^ (*n* = 6)	0.163 ± 0.014 ^a^ (*n* = 23)	0.173 ± 0.018 ^a^ (*n* = 23)
D4	64.82 ± 5.45 ^a^ (*n* = 3)	13.40 ± 1.48 ^a^ (*n* = 9)	13.87 ± 2.38 ^a^ (*n* = 12)	0.160 ± 0.014 ^a^ (*n* = 23)	0.179 ± 0.022 ^a^ (*n* = 23)

Means followed by a different letter in a column are significantly different (†: Tukey’s HSD test and ‡: *t*-test at *p* ≤ 0.05). * Diet: D1, *A*. *franciscana* cysts; D2, A. franciscana cysts plus a vitamin B complex; D3, *A*. *franciscana* cysts plus date palm pollen; and D4, *A*. *franciscana* cysts plus date palm pollen and *E*. *kuehniella* eggs.

**Table 2:** Mean ± SE of the adult longevity and reproductive output of *S. gilvifrons* fed diets containing *A. franciscana* cysts.

* Diet	Male Longevity (Days)	Female Longevity (Days)	Pre- oviposition Period (Days)	Oviposition Period (Days)	Post- oviposition Period (Days)	Total Fecundity (Eggs/fem.)	Egg Hatch (%)	Sex Ratio % fem.
D3	24.92 ± 2.31 ^b^ (*n* = 12)	26.70 ± 2.58 ^b^ (*n* = 10)	3.20 ± 0.49 ^a^ (*n* = 10)	8.50 ± 1.58 ^b^ (*n* = 10)	2.10 ± 0.29 ^a^ (*n* = 10)	33.70 ± 2.38 ^b^ (*n* = 10)	68.17 ± 3.25 ^b^ (*n* = 3)	48.69 ± 3.21 ^a^ (*n* = 3)
D4	27.50 ± 1.09 ^a^ (*n* = 16)	30.80 ± 3.35 ^a^ (*n* = 25)	4.08 ± 0.29 ^a^ (*n* = 25)	12.04 ± 1.45 ^a^ (*n* = 25)	1.56 ± 0.25 ^a^ (*n* = 25)	59.72 ± 3.37 ^a^ (*n* = 25)	80.00 ± 2.88 ^a^ (*n* = 3)	58.72 ± 5.01 ^a^ (*n* = 3)

Means followed by a different letter in a column are significantly different (Student’s *t*-test, *p* ≤ 0.05). * Diet: D3, *A*. *franciscana* cysts plus date palm pollen; and D4, *A*. *franciscana* cysts plus date palm pollen and *E*. *kuehniella* eggs.

**Table 3:** Mean ± SE of the estimated life table parameters of *S. gilvifrons* fed on two different diets containing *A. franciscana* cysts.

* Diet	Parameter					
	*r* (d^−1^)	λ (d^−1^)	*R_o_* (Offspring/fem.)	*GRR* (Offspring/fem.)	*T* (d)	*DT* (d)
D3	0.118 ± 0.015 ^b^	1.125 ± 0.016 ^a^	11.23 ± 2.99 ^b^	26.31 ± 5.53 ^b^	27.47 ± 1.66 ^a^	6.07 ± 1.02 ^a^
D4	0.152 ± 0.007 ^a^	1.162 ± 0.008 ^a^	29.86 ± 4.52 ^a^	46.79 ± 6.75 ^a^	24.34 ± 1.63 ^b^	6.05 ± 0.52 ^a^

The bootstrap procedure was used to calculate the standard errors with 100,000 bootstraps. The means followed by different letters in each column are significantly different between diets using the bootstrap test. * Diet: D3, *A*. *franciscana* plus date palm pollen; and D4, *A*. *franciscana* plus date palm pollen and *E*. *kuehniella* eggs.

## Data Availability

The authors will provide the pertinent data files on ResearchGate.
